# Seasonality of *Coxiella burnetii* among Wild Rabbits (*Oryctolagus cuniculus*) and the *Hyalomma lusitanicum* (Acari: Ixodidae) in a Meso-Mediterranean Ecosystem

**DOI:** 10.3390/pathogens11010036

**Published:** 2021-12-29

**Authors:** María Sánchez, Félix Valcárcel, Julia González, Marta G. González, Raquel Martín-Hernández, José M. Tercero, Pablo González-Jara, A. Sonia Olmeda

**Affiliations:** 1 Villamagna S.A., Finca ‘‘La Garganta’’, 14440 Villanueva de Córdoba, Spain; maria2985@hotmail.com (M.S.); julia.gonzalez@rutgers.edu (J.G.); marta.gonzalez@inia.es (M.G.G.); jmtercero@fincalagarganta.com (J.M.T.); 2 Grupo de Parasitología Animal, Animalario del Departamento de Reproducción Animal, INIA, 28040 Madrid, Spain; pablo.gonzalez@inia.es; 3 Center for Vector Biology, Department of Entomology, Rutgers University, New Brunswick, NJ 08901, USA; 4 Centro Apícola Regional, Bee Pathology Laboratory, JCCM, 19180 Marchamalo, Spain; rmhernandez@jccm.es; 5 Departamento de Sanidad Animal, Facultad de Veterinaria, UCM, 28040 Madrid, Spain; angeles@ucm.es

**Keywords:** *Coxiella burnetii*, *Hyalomma lusitanicum*, wild rabbit, meso-Mediterranean, seasonality

## Abstract

(1) Background: Q fever is a worldwide zoonosis caused by *Coxiella burnetii* that have cases reported in humans and animals almost everywhere. The aim of this study was to describe the seasonality of *Coxiella burnetii* in the wild rabbit (*Oryctolagus cuniculus*) and the tick *Hyalomma lusitanicum* in a meso-Mediterranean ecosystem. (2) Methods: two populations of wild rabbits that differ in whether or not they share habitat with ungulates, mainly red deer (*Cervus elaphus*) were sampled for a year to collect ticks, blood and vaginal or anal swabs. Presence of *C. burnetii* DNA in swabs and the tick *H. lusitanicum* was determined by PCR and serum antibodies by ELISA. (3) Results: *C. burnetii* DNA was detected in 47.2% of 583 rabbits, in 65.5% of sera, and in more than half of the *H. lusitanicum*. There were small variations according to sex and age of the rabbits but significant according to the habitat (4) Conclusions: The results indicate that *C. burnetii* circulates freely between wild rabbits and *H. lusitanicum* and the sylvatic cycle in meso-Mediterranean environments relies in the presence of wild rabbits and *H. lusitanicum* above all if sharing habitat with red deer.

## 1. Introduction

Q fever is a worldwide zoonosis first described in Australia [1] caused by *Coxiella burnetii* (Derrick, 1939; Philip 1948). Except in New Zealand, the infection has been widespread throughout the world, where we find endemic or epidemic areas [2]. It is a notable disease in the United States and the European Union, where it has increased the number of cases reported [3,4].

*Coxiella burnetii* is an obligate intracellular Gram-negative bacterium with two infectious variants, a small cell variant as spore-like form with environmental resistance and a large cell variant that has greater metabolic activity [5]; it can be detected in lung, liver, and spleen in the acute phase of the disease [6]. Besides its importance in Public Health, a broad range of vertebrate and invertebrate hosts, including wild and domestic animals, can be infected [6]. The infection is an important cause of abortion in sheep flocks and it could bring about reproductive losses in red deer in Spain [7,8]. Shedding of *C. burnetii* into the environment occurs mainly during parturition by birth products [9,10], but up to now, several ways of transmission of *C. burnetii* have been reported: ingestion (mainly drinking raw milk), person-to-person transmission, via trans placental, intradermal inoculation, blood transfusion or sexual transmission, although the aerosol route is the primary mode of infection [6]. Little is known about the infection in wildlife [11], in spite of the significant role as reservoir of some wild species, such as rabbits, red deer and small mammals [12,13,14,15].

In addition to all these sources of transmission, ticks are able to spread the pathogen and to be a source of infection [9]. It has been described that one gram of tick faeces contains more than one billion *Coxiella* [16], and that less than ten organisms are capable of causing Q fever [3,17]. The bacterium has been isolated from more than 40 hard tick species, and it was demonstrated the different affinity of Mediterranean ticks for *C. burnetii* in *Dermacentor marginatus* Sulzer, *Rhiphicephalus sanguineus* Latreille, *Rhiphicephalus pusillus* Gil Collado and *Hyalomma lusitanicum* Koch [18]. In consequence, ticks have been suggested to play an important role in the maintenance of *C. burnetii* in nature, as a bridge between wild and domestic animal hosts [2,6,19]. It has only been experimentally confirmed traits related to vector competence in seven tick species (*Dermacentor andersoni* Stiles, *Haemaphysalis bispinosa* Neumann, *Haemaphysalis humerosa* Warburton & Nuttall, *Hyalomma aegyptium* L, *Hyalomma asiaticum* Schulze & Schlottke, *Ixodes holocyclus* Neumann and *R. sanguineus* Latreille) [19].

Some studies suggested the close relationship between *C. burnetii* and wild rabbits in wild meso-Mediterranean environments [7,13,20,21] but the seasonality of this relation has not been described yet. On the other hand, the transstadial transmission of *Hyalomma lusitanicum* ticks from nymph to adults has been recently reported [20]. However, its actual role as a vector of *C. burnetii* remains unknown. Furthermore, in meso-Mediterranean ecosystems the contact between *C. burnetii*, *H. lusitanicum* and susceptible wild animals could occur due to coincidence of the main lambing seasons of wild rabbits and that of red deer. The highest infestation of both host species by immature and/or adults stages of *H. lusitanicum* and the maximum activity of host-seeking adults of this tick specie [21,22,23,24]. Then, the aim of this study was to describe the seasonality of *Coxiella burnetii* in the wild rabbit and the tick *Hyalomma lusitanicum* in two populations of wild rabbits in a meso-Mediterranean ecosystem that differ in whether or not they share habitat with ungulates, mainly red deer.

## 2. Results

Taking into account the overall rabbit population, females were more frequently captured than males (57.3% vs. 42.7%), proportions being marginally non-significant (χ2_(1)_ = 3.69; *p* ≤ 0.055). Regarding the age, most captures were adults (63.5%) and sub adults (24.9%) and just a few young rabbits (11.3%) were sampled, being the relative proportion of captures from each group of age within sex similar in the overall population (χ2_(2)_ = 2.46; *p* ≤ 0.292). Similar sex distribution was observed in both populations although the percentage of adults was higher in the rabbits area (R-A) (74.1%) than in rabbits and ungulates area (RU-A) population (55.2%).

Four tick species were collected: *Rhipicephalus pusillus* Gil Collado 1936, *Hyalomma lusitanicum* Koch 1844, *Haemaphysalis hispanica* Gil Collado 1938 and *Ixodes ventalloi* Gil Collado 1936 (85.9%, 12.9%, 1.0% and 0.2% of the overall collected ticks, respectively), the relative proportion of each species is showed in Table 1. *Rhipicephalus pusillus* was present practically all year round (Supplementary File S1), increasing in numbers from January to May and decreasing thereafter until December, *H. lusitanicum* appeared from May to September and was most abundant in July-August. The other two species *Ha. hispanica* and *I. ventalloi* were present at very low intensity. Remarkably, there were differences between both sampling areas in the majority species found, *R. pusillus* and *H. lusitanicum* (98.4% and 0.3% in R-A and 72.6% and 26.4% in RU-A) (Table 1). When comparisons of the relative proportions of each tick species within area were performed, highly significant differences (χ2_(3)_ = 1435.36; *p* ≤ 0.0001) were found for each of the two major tick species (individual components of χ2_(1)_ ≤ 93 for *R. pusillus* and *H. lusitanicum*), indicating a strong association with area.

*Hyalomma lusitanicum* which is the species of interest in this study was collected in rabbits from June to November in the RU-A (*n* = 1270) whilst in R-A only 15 specimens were found (13 in August and 1 in June and September).

On external inspection of the animals, we found that 96.8% of the animals were healthy and only 3.2% showed some characteristic sign of myxomatosis (bleary eyes, bad general appearance of the fur, etc.) in February, July, August and December. Signs of other diseases were not observed.

*Coxiella burnetii* DNA was detected in 47.2% of 583 swabs analysed, with slightly higher prevalence in females than in males (49.1% and 44.7%) and it was similar at all ages (42.5% to 45.7%), but young females excreted more bacteria than sub-adult and adult females (66.7%, 46.0 and 47.8%, respectively) being differences not significant, χ2_(2)_ = 2.23; *p* ≤ 0.328) (Table 2). When *C. burnetii* prevalence was compared between areas, significant differences were found (χ2_(1)_ = 8.46; *p* ≤ 0.006) being higher in the RU-A than in R-A indicating a strong association with the habitat.

Anti-*C. burnetii* antibodies were detected in 65.5% of sera from wild rabbits tested (*n* = 602) and it was similar in females and males (65.2% and 65.7%) but varied with age. Thus, seroprevalences in adults and sub-adults were significantly higher than in younger individuals (76.7%, 62.0% and 11.43%, respectively, χ2_(2)_ = 112.51; *p* ≤ 0.0001), irrespective of sex (Table 3). Seroprevalence in both study areas followed the same trend in relation to sex and age. Similarly to *C. burnetii* prevalence, seroprevalence was significantly higher (χ2_(1)_ = 12.71; *p* ≤ 0.0004) in rabbits collected in the RU-A than in those collected in the R-A.

It appears that, overall, it was possible to detect the presence of *C. burnetii* DNA in more than half of the *H. lusitanicum* ticks but there were small variations according to sex and age of the rabbits where ticks were collected (Table 4). So, DNA was more frequently detected in *H. lusitanicum* collected from males (58.3%) than from females (51.9%) and from adults (55.3%) than young and sub adults rabbits (50.0% each). The number of *H. lusitanicum* ticks processed in each area of capture was quite different (7 in R-A and 171 in RU-A) so it is difficult to make a direct comparison. Nevertheless *C. burnetii* detection in both situations was over 40% of the sampled *H. lusitanicum* (42.9% in R-A and 55.0% in RU-A).

There were apparently no major differences between males and females in the seasonal pattern of antibody or DNA excretion (Supplementary Files S2 and S3). The dynamics of DNA excretion in sub-adult wild rabbits was higher than in adults but it was the opposite in relation to antibodies that were lower and more fluctuant in sub-adults than in adults; unfortunately, the number of young wild rabbits sampled was not sufficient to draw reasonable conclusions, but it appears that they become infected in April and release antibodies in May, with both levels increasing thereafter (Supplementary Files S4 and S5).

**Figure 1:**
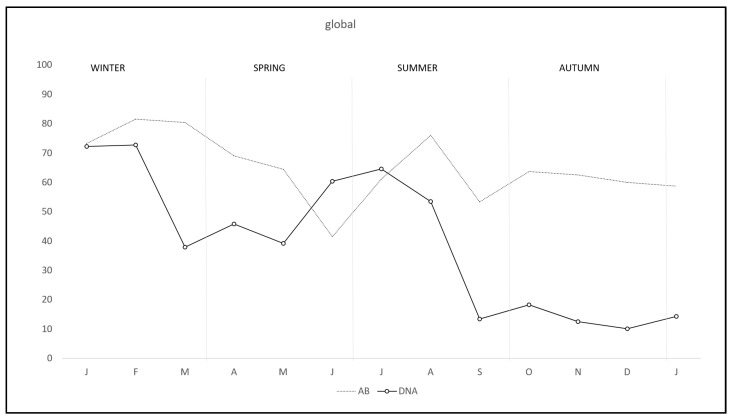
Monthly overall prevalence of *C. burnetii* DNA excretion and anti-*C. burnetii* antibodies in wild rabbits in a meso-Mediterranean ecosystem.

**Figure 2:**
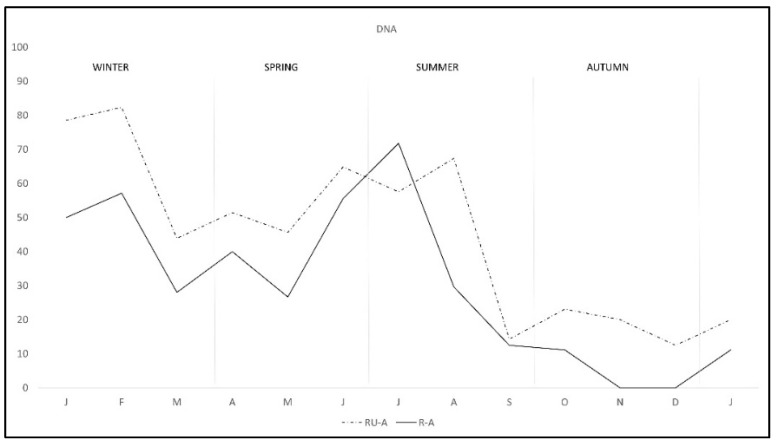
Monthly *C. burnetii* DNA excretion in two wild rabbit populations in a Meso-Mediterranean ecosystem. R-A = rabbits and ungulates area, RU-A = non ungulates area.

During the study there was a clear overall pattern of *C. burnetii* DNA excretion and subsequent antibody production (Figure 1). In the first two months of the study—winter—72.7% of the rabbits excreted DNA, decreasing from March to May—spring—and rising again from June to August—summer—and finally decreasing to the lowest levels from September to January—autumn-winter. Correspondingly antibody pattern was similar but lagged some weeks behind DNA excretion until September, when the antibody level remained established. Interestingly, the pattern of DNA excretion in both study areas was similar, although monthly percentage in R-A was lower than in RU-A along the study except in July (Figure 2). 

**Figure 3:**
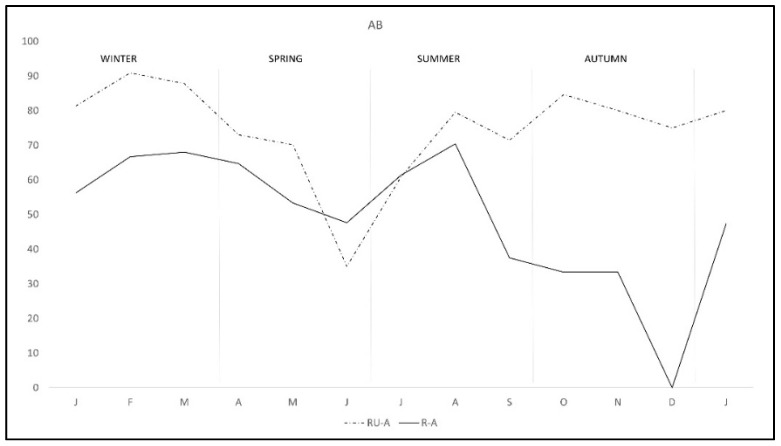
Monthly production of anti-*C. burnetii* antibodies in two wild rabbit populations in a meso-Mediterranean ecosystem. R-A = rabbits and ungulates area, RU-A = non ungulates area.

**Figure 4:**
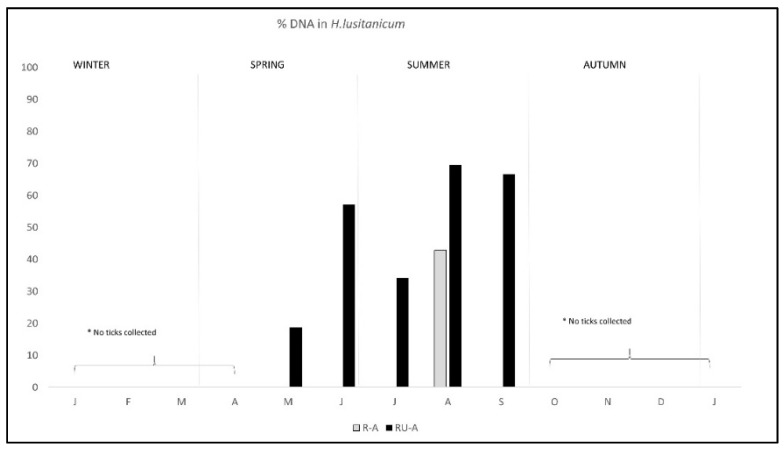
Monthly percentage of *C. burnetii* DNA detection/presence in *Hyalomma lusitanicum* ticks from two wild rabbit populations in a meso-Mediterranean ecosystem. R-A = rabbits and ungulates area, RU-A = non ungulates area. * months in which we could not collect *H. lusitanicum*.

The pattern of antibodies in R-A was quite similar in both areas from January to August, with lower percentage in R-A than in RU-A, but from September to December seroprevalence varied with the area, progressively decreasing in R-A while it was maintained in rabbits from RU-A (Figure 3). We only found DNA in *H. lusitanicum* from May to September and practically only in RU-A since in R-A the collection of *H. lusitanicum* was very low (Figure 4).

## 3. Discussion

The high number of wild rabbits captured confirms its adaptation to the meso-Mediterranean conditions from which it originated [25,26]. Rabbit females were captured more frequently than males during the time prior to parturition, when females spend more time outside the burrows in search of food and males. In contrast, more males were captured from June to September and in December, when females remain mostly in the burrows tending their litters. This sex ratio is the habitual situation in wild rabbits population in this ecosystem, where sex ratio is usually balanced or slightly biased towards females [27]. Age structure of captures (more than 90% sub-adults and adults) also represents a typical wild population in which new born rabbits stay in the burrow for approximately 20 days before they start exploring the field and then, due to their rapid maturation, quickly move into the older age groups. Adults and sub-adults were captured throughout the year but the youngest individuals (<0.8 kg) were captured from April to July and in January. Thus, a long and abundant breeding season was estimated between February and June and a smaller one at the end of the year. This reproductive period coincides with the usual one in these areas (between November and June) and is influenced by local conditions of temperature and rainfall intensity [28,29,30].

During years prior to the study the rabbit population in the area used to drop sharply in September due to myxomatosis (personal observations). This previous situation is in agreement with [31] who reported a “seasonal gap” causing a decrease in the rabbit population in late summer due to viral diseases. Nevertheless, the mean seroprevalence during the year of study of myxomatosis and rabbit haemorrhagic disease were 88% and 77% with antibodies against both viruses in 70% of the rabbits [32]. Then, it is assumed that rabbit population was reasonable protected by antibodies as it is shown by the healthy status of rabbits observed in external inspection. Apart from diseases, the main cause of death is predation, above all in juveniles and sub-adults [28,33,34,35], but this factor is constant throughout the year in the study areas (personal observations) so it should not be the cause of the decrease in captures. It is most likely that the reduction in the number of captures in these last months is due to the fact that the rabbits have learnt to avoid the fence where they are collected.

Due to the methodological design we could not establish a direct comparison with other studies on the number of ticks per rabbit because we only sampled those ticks attached to the ears of the rabbits thus we underestimated the total tick index. Nevertheless, the abundance of *Rhipicephalus pusillus* and *Hyalomma lusitanicum* are in agreement with the studies of other authors [21,22,23,24,36] which reported that they are the commonest tick species in this and other areas of the Iberian Peninsula while *Haemaphysalis hispanica* and *Ixodes ventalloi* being also typical of wild rabbits do not usually appear with such intensity. González et al. [21] indicated that *H. lusitanicum* is the predominant species in wild rabbits in this ecosystem above all in spring and summer and *R. pusillus* is present all the year in lower amounts. In present study *R. pusillus*—all stages—was the predominant specie and was found along all the year whilst *H. lusitanicum*—immature stages—was mainly found in summer. The differences were due to the effect of trapping location which strongly influenced tick populations by changing host abundance [37]. So, tick population in RU-A is in agreement with previous studies in this ecosystem where red deer and wild rabbits are the main host of *H. lusitanicum* adults as revised by Valcárcel et al. [38]. In contrast, the absence of ungulates at R-A limited the abundance of *H. lusitanicum*.

The inhalation of birth products is the main route of infection of Q fever; in consequence, it is presumed that ticks play a minor role in Q fever transmission in the domestic cycle but they may play a role as the natural reservoir in the sylvatic cycle [2] where transmission between arthropods and wild animals should be considered [39].

It was reported that most soft and hard ticks are able to transmit *C. burnetii* transstadially and it was thought that most of them could do it transovarially [40]. However, because of the different results about the presence of *C. burnetii* in several tick species, the consideration of tick abundance as a risk factor for Q fever is controversial. Some studies found no significant correlations due to the low global prevalence of *C. burnetii* in ticks (<5%) [41] and others did find an association with the abundance ticks [2,42,43]. The differences between the research findings are probably due to the species of ticks and the ecological particularities of the different areas where the studies were carried out. In the present study, *C. burnetii* DNA was detected in more than 50% of *H. lusitanicum*, a similar percentage to that reported by González et al. [22], suggesting that it had increased in central Spain compared to just two decades ago with a reported prevalence of 10% in Spain and Portugal [41,44] and much higher than in other tick species [41].

Some ticks can carry a pathogen without being able to transmit it [45]. Although the most abundant tick species found in our work were of the genus *Rhipicephalus*, we focused on *H. lusitanicum* because of its known role in the transmission of *C. burnetii* and the high presence of this bacterium in this tick species [20,22]. In fact, we were able to detect *C. burnetii* from *H. lusitanicum* collected from both infected and uninfected wild rabbits confirming previous results in this species [20,22] as already reported in other tick species [46]. The absence of experimental studies about transmission is a great inconvenient to know the real role of *Hyalomma lusitanicum* to transmit *C. burnetii*. However, there are field evidences in both studies of González et al. about the adaptation of the bacteria to the pass from one stage to the next of naturally infected *H. lusitanicum* and that at least transstadial transmission is happening in meso-Mediterranean environments. In addition, DNA can also be detected in 33% of the batches of offspring from engorged females, suggesting transovarial transmission; unfortunately, in both studies the viability of the bacteria was not tested.

The presence of *C. burnetii* in wild rabbit has also been found with higher prevalence where host density is greater as it happens in our study [13,21,39]. Sex influenced the excretion of *C. burnetii* DNA, which was slightly higher in adult females than in adult males due to the high concentration of bacteria in birth products despite the fact that it can also be excreted in faeces, urine, and milk of infected animals [2,47]. Our results seem to indicate that anal swabs could also be of similar value to vaginal swabs in detecting *C. burnetii* prevalence in wild rabbits.

Other studies suggested the essential role of wild rabbits in the maintenance of the bacteria in wildlife environments [13,22]. We agree with these authors and the results of seasonality of both antibodies and DNA support that the bacteria circulate in this host all around the year with small differences due to the age, sex and the location of captures.

DNA detection was similar at all ages; however, seral prevalence increased with age, as younger rabbits had less opportunity to become infected than sub-adults and adults. The DNA presence in vaginal swaps of young females was higher than that of sub-adult and adult females, but their seroprevalence was low, probably because at the time of sampling they were freshly infected and did not have time to develop antibodies. Therefore, it is reasonable to conclude that the first infection occurs early and, consequently, as the immune response develops, bacterial shedding decreases. Control of the first *C. burnetii* infection is primarily due to the Th1 response and IFN production while antibodies are dispensable [2]. Consequently, the reduction in DNA excretion should be due to a successful Th1 response with a consequent decrease in antibodies that might serve as an indicator of the curation. We have not found references on the duration of anti-*C. burnetii* antibodies, however the regular decline of antibodies after DNA depletion may indicate that they are short-lived.

It would be expected that DNA excretion and antibody production would be similar in both areas throughout the year, but this only happened during the first nine months of the study. In the beginning there should be a finishing active infection in which immune responses cause DNA excretion to decrease until May, rabbits should not be re-infected in these first months because there was a posterior antibody reduction. Later, a new infection coinciding with the main spring breeding season occurred with an increase in DNA and subsequent antibodies. In summer and autumn, rabbits spend more time out of the burrow and there is little or no reproduction, so the re-infection chance among rabbits is low in R-A, which explains the reduced DNA and antibodies in September that remained in low levels since then. However, from September to January antibody production remained at high levels in rabbits from RU-A despite a similar decrease in DNA prevalence. In this point it is needed to note that the number of captures at the end of the study was very low and results should be carefully considered. The differences could be due to the existence of an antigenic stimuli in RU-A sufficient to stimulate the immune response by producing antibodies. This antigenic stimulus could have two origins, the progression of persistent *C. burnetii* infection, reflecting a failure of the Th1 response, or it may be due to weak reinfections. We cannot rule out persistent infections even though we have not found any evidence of altered health status and it is supposed to occur in both areas. However, it is possible the reinfection through bacteria excreted by red deer or transmission by *H. lusitanicum* ticks.

Wild rabbits and red deer share habitat in the RU-A so it is possible that wild rabbits are infected by a spore-like form bacteria excreted during red deer calving in spring or in September-October during the mating call of red deer because *C. burnetii* can survive long periods in soil due as resistant spore-like forms [48] and they can be easily transported by the wind [49]. This possibility is much lower in the R-A because of the absence of red deer.

*Coxiella burnetii* is multiplicity primarily inside gut epithelial cells of other tick species remaining viable inside the tick’s body between 200, in *Hyalomma aegyptium* [50] and 1000 days in *Ornithodoros turicata* [51]. Furthermore, depending on the tick species and faeces, it can contain 10^3^ to 10^8^ viable bacteria for up to 635 days [2]; it can therefore be transmitted directly by the tick’s saliva during feeding or indirectly through infected faeces using damaged skin as a route of entry. There is no data about survival of *C. burnetii* in *H. lusitanicum* but transmission through saliva has been recently reported in artificial feeding [20]. These authors did not find the bacterium in *H. lusitanicum* faeces but do not rule out the possibility. If confirmed, it would be a serious source of infection of rabbits as ear damage from fighting rabbits is very common (Supplementary File S6). The high prevalence of *H. lusitanicum* in red deer and wild rabbits in RU-A suggests that this tick species may bridge the gap between rabbits and red deer and has an important epidemiological role in the sylvatic cycle of *C. burnetii* in meso-Mediterranean environments.

## 4. Materials and Methods

### 4.1. Study Area

The study was carried out in a private natural reserve situated in Ciudad Real (Central Spain: 38°26′35.65′′ N; 4°29′34.31′′ E). The site covers an area of 13,000 ha at a mean altitude of 669 m above sea level; annual average rainfall is 650 mm and temperatures range from −4 to 43 °C. This reserve harbours a wild fauna and flora representative of the meso-Mediterranean bioclimatic environment (a more extensive description can be seen in González et al. (2016)).

For the sampling we selected two sampling areas which were separated 20 years ago by a hunting fence. Both areas had similar flora and fauna, and there are available feeders and drinkers near to the burrows, which are checked daily by the farm staff for proper functioning. The first one is an area of 540 ha where wild rabbits and ungulates share habitat (RU-A: rabbits and ungulates area) whilst in the other area, 340 ha, there is no presence of ungulates (R-A: no ungulates area or rabbits area) (for more details see Valcárcel et al. [37]. To ensure a comparable sampling efforts and number of catches, they were carried out at five and four sampling points, respectively.

### 4.2. Host Sampling and Management

The study was conducted along 13 months, wild rabbits were monthly captured using a traditional method commonly used to ensure animal welfare. The technique consisted of placing a rabbit netting 15 days before sampling between the planting area, where the rabbits feed, and the bush, where they have their burrows. The mesh, 1 m high, was fixed to the ground by means of pegs driven into its base (bent about 25–30 cm in the direction of planting) except for certain areas, “visors” which remained open for the rabbits to familiarise themselves with and pass through on a daily basis. The netting was not laid in a linear arrangement, but formed “shelter pockets” every 20–30 m. In the early morning of the day of the capture, when the rabbits are in the sowing area, the “visors” are lowered and the netting is firmly fixed to the ground. When the rabbits try to return to their burrows, they find the path closed and take refuge in the shelter pockets and are all collected by hand by the farm’s qualified personnel, who place them in authorised transport cages.

After capture wild rabbits were quickly transported to the laboratory located in the same reserve. Immediately after identification and data and sample collection, animals were transported again to the same sampling point where they were captured. Captures, management and sample collection was performed with the authorization of the Animal Ethic Committee of the Regional Government (PROEX 14-2018 “Monitorización del estado sanitario del conejo de campo”) to preserve animal welfare in accordance with the general conditions for the housing and care of animals set out in Article 6 of Royal Decree 53/2013 of 1 February, which establishes the basic rules applicable to the protection of animals used for experimental and other scientific purposes, including teaching.

Each rabbit was handled individually in the laboratory. Firstly, they were weighed with a portable scale (Cobos precision model CR-20^®^), sexed and identified by placing metal ear tags (Hauptner^®^). The age of the animals was established based on live weight and assigned to one of the three groups of age as follows: young <800 g, subadult = 800–1100 g and adult = > 1100 g) [52]. Subsequently, an external inspection was carried out to determine whether there were clinical signs of any pathology, in particular the clinical signs of myxomatosis and rabbit haemorrhagic disease.

To maintain rabbit welfare by reducing handling time, only those ticks attached to ears were picked up with fine tweezers and were individually stored in labelled Eppendorf^®^ tubes with 70% alcohol until identification by specific keys [53,54,55,56,57]. Ear parasite index of ticks were recorded (EPI = number of ticks collected in rabbit´s ears).

A 0.5 mL sample of blood was collected from marginal ear vein and preserved in tubes without anticoagulant. Vaginal or anal samples from females and males were respectively removed with sterile swabs. Ticks and swaps were stored at −20 °C prior DNA extraction and blood samples were stored at 4 °C prior ELISA testing.

### 4.3. Preparation of Samples for PCR Analysis

Ticks were 70%-ethanol-disinfected and then adult ticks were cut with scissors to assist DNA extraction. Specimens of *H. lusitanicum* were individually processed and placed into a well of a 96-well collection plate (QIAGEN, Hilden, Germany). Each vial included 3 stainless steel beads (3 mm) (Werfen, Barcelona, Spain) and 200 μL miliQ water. Plates were sealed and shaken in a TissueLyser machine (QIAGEN, Hilden, Germany) at 30 Hz (2 cycles of 6 min) for disruption and homogenization. After this, every sample (150 µL) were mixed with a solution containing 20 µL of proteinase K (QIAGEN, Hilden, Germany) and 30 µL of ATL buffer (QIAGEN, Hilden, Germany); then the mix was kept at 56 °C overnight stirring at 900 rpm.

Vaginal and anal swabs were also individually put into a well of the collection plate with a solution containing 20 µL of proteinase K and 400 µL of ATL buffer, then were kept at 56 °C overnight stirring at 900 rpm.

### 4.4. PCR Analysis

DNA was extracted using BS96 DNA Blood Kit (QIAGEN, Hilden, Germany) following the protocols for tissues (ticks) or swabs respectively in a BioSprint 96 workstation (QIAGEN, Hilden, Germany). More detailed descriptions can be seen in González et al. [20,21].

Cross-contamination during DNA extraction was excluded by running in parallel negative controls (nuclease-free water; GE Healthcare Life Sciences, Logan, UT, USA); one negative control every 20 samples was included per plate. We used a *C. burnetii* positive control kindly supplied by Institute of Health Carlos III (strain Nine Mile phase II). We evaluated a sample to be positive until a threshold cycle (C_T_) value 40. All controls were negative and *C. burnetii* positive controls presented their corresponding positive results and did not show any cross-reaction.

### 4.5. ELISA Analyses

Blood samples were centrifuged to obtain serum for subsequent serological testing by performing an indirect ELISA assay for *Coxiella burnetii* using the commercial kit LSIVetTMRuminant Q Fever-Serum/Milk (Life Technologies^TM^, Waltham, MA, USA) according to the manufacturer’s instructions. Basically, “samples and controls are spread on the plate coated with *C. burnetii* antigen, where specific anti-*C burnetii* antibodies bind to the antigen. After washing, a peroxidase-labelled G-protein conjugate (HRP) is added which binds to the antibodies previously attached to the plate. The unbound conjugate is removed by washing, followed by the addition of a chromogenic substrate. Oxidation of the substrate by the HRP conjugate produces a blue colour. After stopping the reaction, the colour turns yellow and the results are then read using an ELISA plate reader. The intensity of the yellow colour present in positive samples is proportional to the amount of specific antibody in the sample.

The S/P (Sample/Positive) ratio is calculated for each sample by calculating the mean OD (Optical Density) of the positive control (ODm PC), and that of the negative control (ODm NC)”.

S/P = (OD sample−ODm NC)/(ODm PC−ODm NC)

Titer = S/P × 100

### 4.6. Data Analyses

In the first month (January) we performed a strong effort to assure an enough number of rabbits (*n* = 284) needed to know the starting situation and to get the chance of recapturing them along the year. In the following twelve months (February to January) a total of 727 wild rabbits were captured then a total of 1011 captures were performed along the thirteen months of the study. A total of 149 wild rabbits were recaptured two or more times (total captures of them = 347). As the aim of the study was to obtain a global overview and a seasonal description of the prevalence of *C. burnetii* in rabbits and its percentage in *H. lusitanicum* ticks, recaptured rabbits were only considered once (the first capture) in the data processing.

A goodness of fit tests was used to analyse the relative proportion of each tick species upon the area. Similarly, to compare the prevalence of *C. burnetii* and the prevalence of antibodies against *C. burnetii* in the rabbit population, goodness-of-fit tests were performed, considering comparisons between areas, comparisons between age groups within each area and comparisons between sexes within each area. Yate´s correction for continuity was applied for all 2 × 2 comparisons performed.

## 5. Conclusions

This study confirms that *C. burnetii* circulates freely between wild rabbits and *Hyalomma lusitanicum* and the sylvatic cycle relies in the presence of them in meso-Mediterranean environments where they are present. This circulation occurs all around the year with significant difference according to the habitat (red deer presence) but no large differences between wild rabbit sex with apparently more DNA excretion by rabbits during breeding season and that bacteria harboured by *H. lusitanicum* is enough to maintain and transmit the bacteria. Further studies are needed to determine the viability of the bacterium in the different stages and faeces of *H. lusitanicum*, as well as the role of other tick species and micromammals in its maintenance in wildlife and possible connections with the domestic cycle.

## Figures and Tables

**Table 1:** Main descriptive parasitation parameters of tick infestation in two wild rabbit populations in a meso-Mediterranean ecosystem. RU-A = rabbits and ungulates area, R-A = non ungulates area or rabbit area. EPI = ear parasite index. % expresses the proportion of tick species.

Area (Rabbits)	EPI	*Rhipicephalus*	*Hyalomma*	*Haemaphysalis*	*Ixodes*	Ticks
R-A (265)	*n*	5.027	15	53	12	5.107
	Mean	18.97	0.06	0.20	0.05	19.27
	Variance	640.84	0.26	0.42	0.12	644.88
	Median	7	0	0	0	8
	%	98.43	0.29	1.04	0.23	100.00
RU-A (344)	*n*	3.496	1.270	43	8	4.817
	Mean	7.21	3.69	0.09	0.02	9.93
	Variance	138.01	141.58	0.23	0.06	257.08
	Median	3	0	0	0	4
	%	72.58	26.36	0.89	0.17	100.00
Overall (609)	*n*	8.523	1.285	96	20	9.924
	Mean	10.46	2.11	0.12	0.02	12.18
	Variance	375.16	83.24	0.31	0.09	431.83
	Median	4	0	0	0	6
	%	85.88	12.95	0.97	0.20	100.00

**Table 2:** Overall prevalence of *Coxiella burnetii* DNA in two wild rabbit populations in a meso-Mediterranean ecosystem.

Sex and Age	Global	R-A	RU-A
	%	%	%
Female	49.09 (162/330)	43.15 (63/146)	53.80 (99/184)
Young	66.67 (20/30)	66.67 (8/12)	66.67 (12/18)
Subadult	45.95 (34/74)	44.00 (11/25)	46.94 (23/49)
Adult	47.79 (108/226)	40.37 (44/109)	54.70 (64/117)
Male	44.66 (113/253)	35.85 (38/106)	51.02 (75/147)
Young	45.71 (16/35)	33.33 (4/12)	52.17 (12/23)
Subadult	42.65 (29/68)	18.75 (3/16)	50.00 (26/52)
Adult	45.33 (68/150)	39.74 (31/78)	51.39 (37/72)
Total	47.17 (275/583)	40.08 (101/252)	52.57 (174/331)
Young	55.38 (36/65)	50.00 (12/24)	58.54 (24/41)
Subadult	44.37 (63/142)	34.15 (14/41)	48.51 (49/101)
Adult	46.81 (176/376)	40.11 (75/187)	53.44 (101/189)

The presence of DNA was estimated by PCR in swabs (collected from anus in males or vagina in females). RU-A = rabbits and ungulates area, R-A = non ungulates area.

**Table 3:** Overall prevalence of Anti-*C. burnetii* antibodies in two wild rabbit populations in a Meso-Mediterranean ecosystem.

Sex and Age	Global	R-A	RU-A
	%	%	%
Female	65.22 (225/345)	57.05 (89/156)	71.96 (136/189)
Young	14.29 (2/14)	14.29 (2/14)	14.29 (3/21)
Subadult	56.41 (7/25)	28.00 (7/25)	69.81 (37/53)
Adult	75.86 (80/117)	68.38 (80/117)	83.48 (96/115)
Male	65.76 (62/107)	57.94 (62/107)	71.33 (107/150)
Young	8.57 (2/12)	16.67 (2/12)	4.35 (1/23)
Subadult	68.06 (6/17)	35.29 (6/17)	78.18 (43/55)
Adult	78.00 (54/78)	69.23 (54/78)	87.50 (63/72)
Total	65.45 (151/263)	57.41 (151/263)	71.68 (243/339)
Young	11.43 (4/26)	15.38 (4/26)	9.09 (4/44)
Subadult	62.00 (13/42)	30.95 (13/42)	74.07 (80/108)
Adult	76.70 (134/195)	68.72 (134/195)	85.03 (159/187)

The presence of antibodies was estimated in serum samples by ELISA. R-A = rabbits and ungulates area, RU-A = non ungulates area.

**Table 4:** Overall prevalence of *Coxiella burnetii* DNA in *Hyalomma lusitanicum* collected in two wild rabbit populations in a Meso-Mediterranean ecosystem. The presence of DNA in ticks was individually estimated by PCR. R-A = rabbits and ungulates area, RU-A = non ungulates area.

Sex and Age Where *H. lusitanicum* Were Collected	Global	R-A	RU-A
	%	%	%
Female	51.89 (55/106)	20.00 (1/5)	53.47 (54/101)
Young	-	-	-
Subadult	40.00 (4/10)	-	40.00 (4/10)
Adult	53.13 (51/96)	20.00 (1/5)	54.95 (50/91)
Male	58.33 (42/72)	100.00 (2/2)	57.14 (40/70)
Young	50.00 (1/2)	-	50.00 (1/2)
Subadult	56.25 (9/16)	-	56.25 (9/16)
Adult	59.26 (32/54)	100.00 (2/2)	57.69 (30/52)
Total	54.49 (97/178)	42.86 (3/7)	54.97(94/171)
Young	50.00 (1/2)	-	50.00 (1/2)
Subadult	50.00 (13/26)	-	50.00 (13/26)
Adult	55.33 (83/150)	42.86 (3/7)	56.34 (80/142)

## Data Availability

Not applicable.

## Supplementary Materials

The following supporting information can be downloaded at: https://www.mdpi.com/article/10.3390/pathogens11010036/s1, Supplementary file S1: Monthly collection of ticks in the ears of two wild rabbit populations in a meso-Mediterranean ecosystem. R-A = rabbits and ungulates area, RU-A = non ungulates area; Supplementary file S2: Monthly production of anti-C. *burnetii* antibodies in wild rabbits in a meso-Mediterranean ecosystem; Supplementary File S3: Monthly C. *burnetii* DNA excretion in wild rabbits in a meso-Mediterranean ecosystem; Supplementary file S4: Monthly percentage of C. *burnetii* DNA excretion in wild rabbits in a meso-Mediterranean ecosystem according to the age; Supplementary file S5. Monthly percentage of antibodies anti-C. *burnetii* in wild rabbit in a meso-Mediterranean ecosystem according to the age; Supplementary file S6. Ear damage from fighting rabbits is very common.
