# Supplementary material for: Seasonality of Coxiella burnetii among Wild Rabbits (Oryctolagus cuniculus) and the Hyalomma lusitanicum (Acari: Ixodidae) in a Meso-Mediterranean Ecosystem
Source: Pathogens. 2021 Dec 29;11(1):36. doi: 10.3390/pathogens11010036 (PMC8781871; doi:10.3390/pathogens11010036)
Supplement: Supplementary file 1 [file pathogens-11-00036-s001.zip › pathogens-1532309-supplementary.pdf]

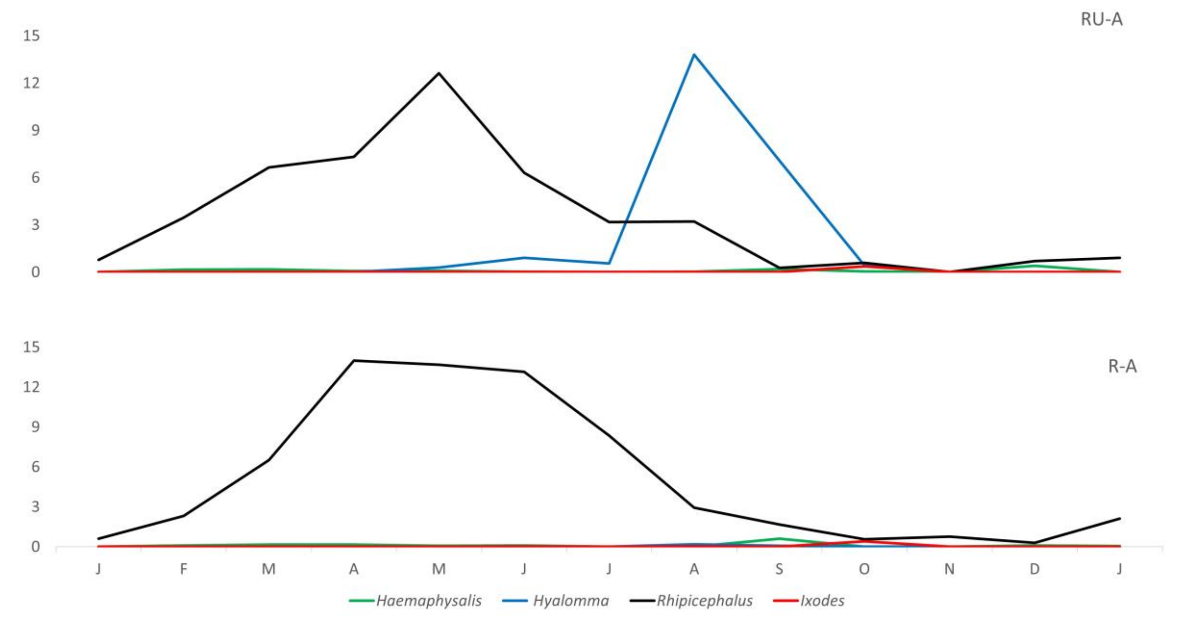

Supplementary file S1: Monthly collection of ticks in the ears of two wild rabbit populations in a meso-Mediterranean ecosystem. R-A = rabbits and ungulates area, RU-A = non ungulates area.

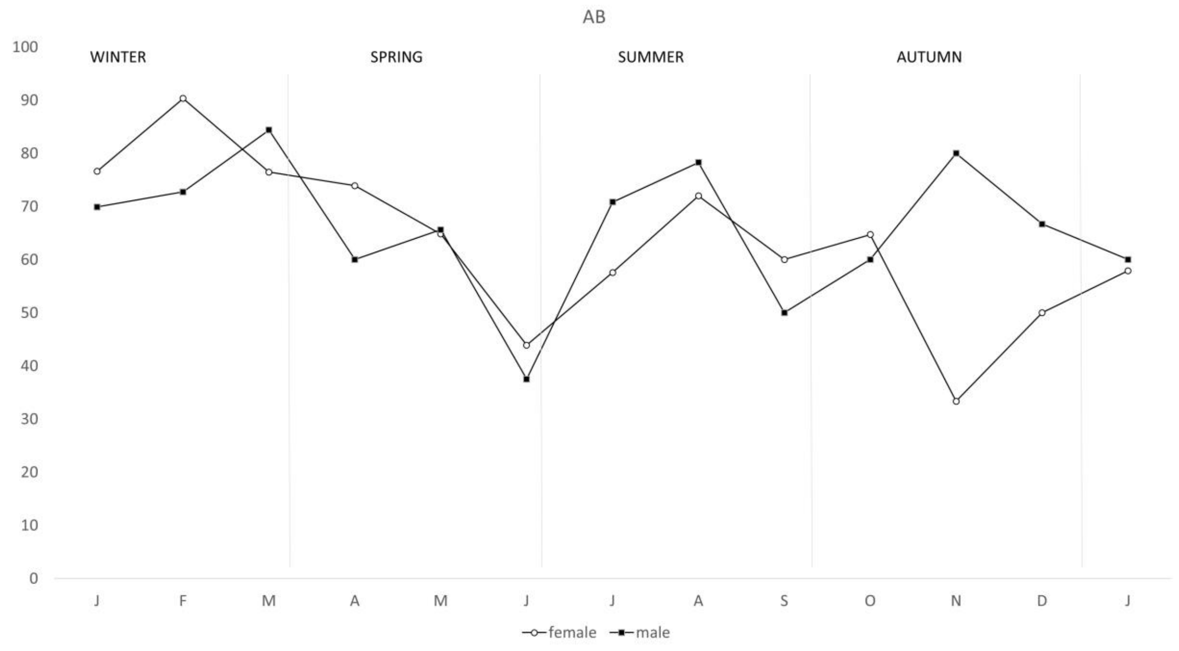

Supplementary file S2: Monthly production of anti-C. *burnetii* antibodies in wild rabbits in a meso-Mediterranean ecosystem.

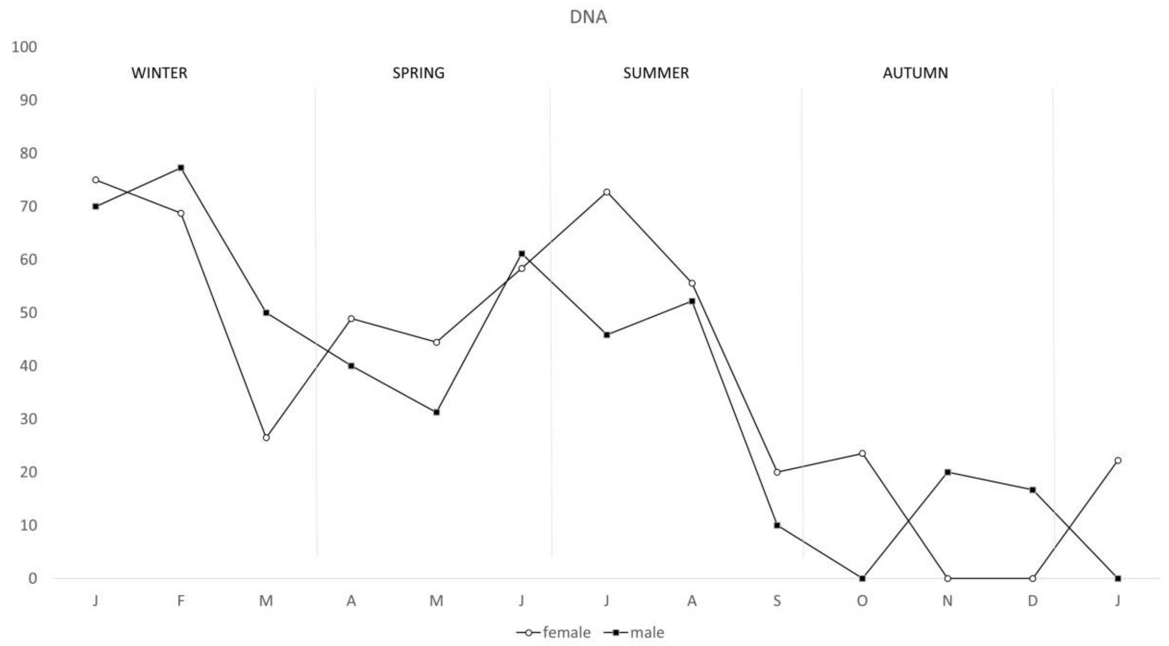

Supplementary File S3: Monthly C. *burnetii* DNA excretion in wild rabbits in a meso-Mediterranean ecosystem.

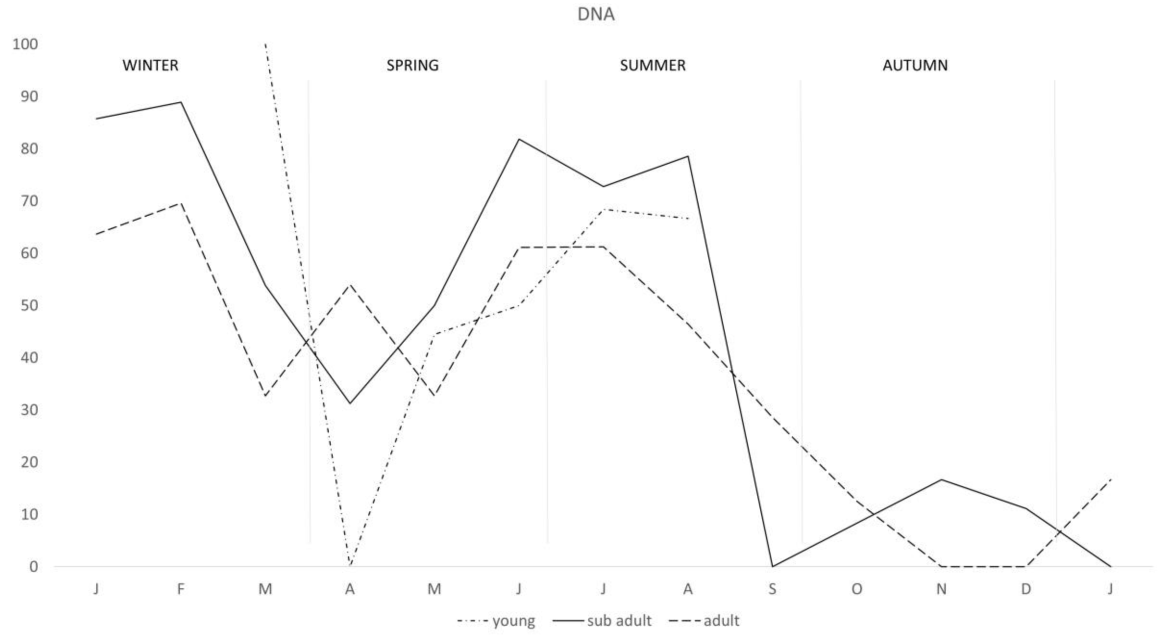

Supplementary file S4: Monthly percentage of C. *burnetii* DNA excretion in wild rabbits in a meso-Mediterranean ecosystem according to the age.

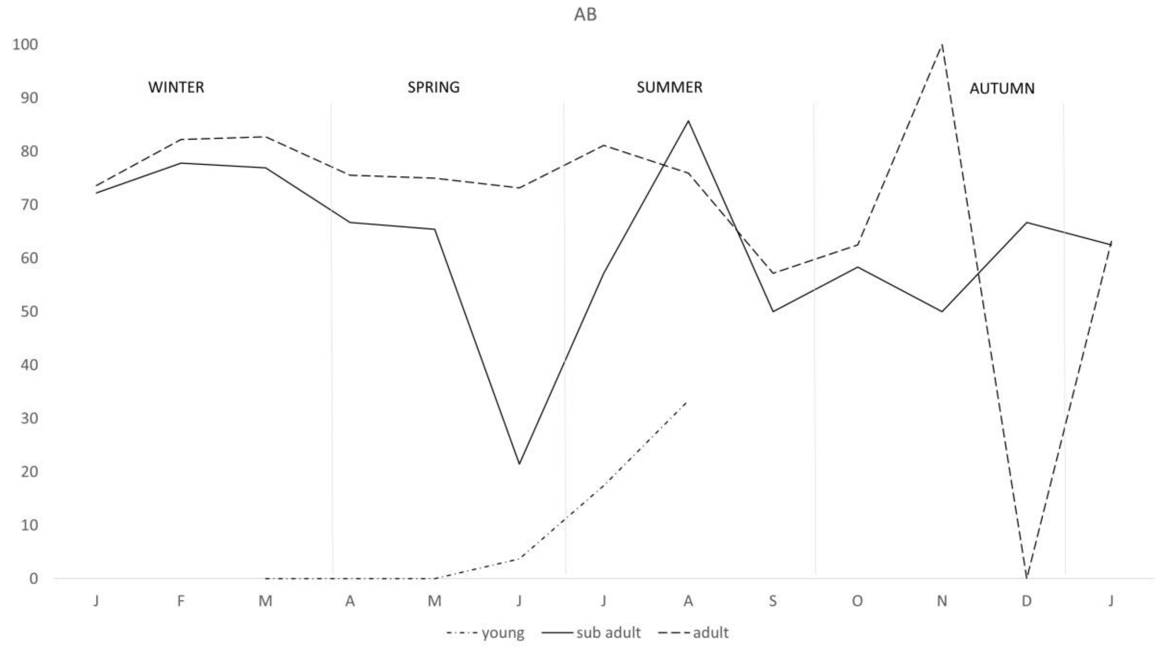

Supplementary file S5. Monthly percentage of antibodies anti-C. *burnetii* in wild rabbit in a meso-Mediterranean ecosystem according to the age.

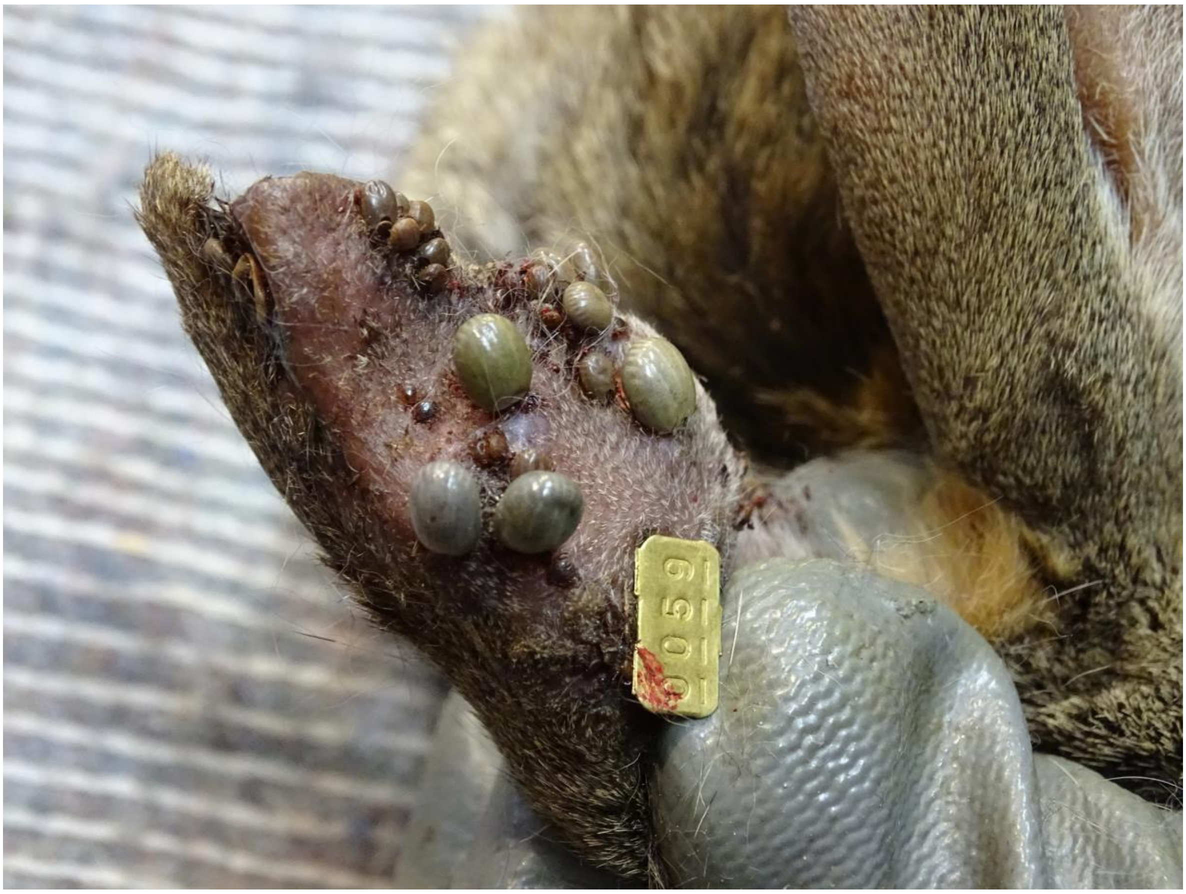

Supplementary file S6. Ear damage from fighting rabbits is very common.
